# Novel Intraoperative Navigation Using Ultra-High-Resolution CT in Robot-Assisted Partial Nephrectomy

**DOI:** 10.3390/cancers14082047

**Published:** 2022-04-18

**Authors:** Kiyoshi Takahara, Yoshiharu Ohno, Kosuke Fukaya, Ryo Matsukiyo, Takuhisa Nukaya, Masashi Takenaka, Kenji Zennami, Manabu Ichino, Naohiko Fukami, Hitomi Sasaki, Mamoru Kusaka, Hiroshi Toyama, Makoto Sumitomo, Ryoichi Shiroki

**Affiliations:** 1 Department of Urology, Fujita-Health University School of Medicine, Nagoya 470-1192, Japan; kofukaya@fujita-hu.ac.jp (K.F.); takuhisa@fujita-hu.ac.jp (T.N.); masashi.takenaka@fujita-hu.ac.jp (M.T.); zenken@fujita-hu.ac.jp (K.Z.); michino@fujita-hu.ac.jp (M.I.); sasakih@fujita-hu.ac.jp (H.S.); m-sumi@fujita-hu.ac.jp (M.S.); rshiroki@fujita-hu.ac.jp (R.S.); 2 Department of Radiology, Fujita-Health University School of Medicine, Nagoya 470-1192, Japan; yohno@fujita-hu.ac.jp (Y.O.); rie-haya@fujita-hu.ac.jp (R.M.); htoyama@fujita-hu.ac.jp (H.T.); 3 Department of Urology, Okazaki Medical Center, Fujita Health University, Okazaki 444-0827, Japan; fukami-n@fujita-hu.ac.jp (N.F.); mkusaka@fujita-hu.ac.jp (M.K.)

**Keywords:** estimated blood loss, robot-assisted partial nephrectomy, ultra-high-resolution computed tomography, warm ischemia time

## Abstract

**Simple Summary:**

Successful surgery in robot-assisted partial nephrectomy (RAPN), especially for highly complex tumors, relies on a detailed understanding of the anatomical relations of the tumor absolute and relative to the urinary tract and the vascular structures, including the renal pedicle. Intraoperative navigation with accurate information regarding tumor position relative to the surrounding urinary vascular structures undoubtedly assists the surgeon during RAPN. In this report, we performed RAPN with intraoperative navigation using a novel computed tomography scanner (UHR-CT) and compared its perioperative and short-term functional outcomes to those of area-detector CT (ADCT). We found that this novel navigation system using UHR-CT provided a shorter warm ischemia time and lower estimated blood loss than ADCT, and concluded this could be a useful tool for patients who undergo RAPN. This is the first report to evaluate the feasibility and usefulness of UHR-CT for intraoperative navigation during RAPN.

**Abstract:**

To assess the perioperative and short-term functional outcomes of robot-assisted partial nephrectomy (RAPN) with intraoperative navigation using an ultra-high-resolution computed tomography (UHR-CT) scanner, we retrospectively analyzed 323 patients who underwent RAPN using an UHR-CT or area-detector CT (ADCT). Perioperative outcomes and the postoperative preservation ratio of estimated glomerular filtration rate (eGFR) were compared. After the propensity score matching, we evaluated 99 patients in each group. Although the median warm ischemia time (WIT) was less than 25 min in both groups, it was significantly shorter in the UHR-CT group than in the ADCT group (15 min vs. 17 min, *p* = 0.032). Moreover, the estimated blood loss (EBL) was significantly lower in the UHR-CT group than in the ADCT group (33 mL vs. 50 mL, *p* = 0.028). However, there were no significant intergroup differences in the postoperative preservation ratio of eGFR at 3 or 6 months of follow-up (ADCT 91.8% vs. UHR-CT 93.5%, *p* = 0.195; and ADCT 91.7% vs. UHR-CT 94.0%, *p* = 0.160, respectively). Although no differences in short-term renal function were observed in intraoperative navigation for RAPN in this propensity score–matched cohort, this study is the first to demonstrate that UHR-CT resulted in a shorter WIT and lower EBL than ADCT.

## 1. Introduction

Partial nephrectomy (PN) for small renal tumors is preferred over radical nephrectomy with respect to surgery-related mortality, cancer-specific survival, time to recurrence, and renal function [1,2]. Over the past decade, significant advantages of robot-assisted PN (RAPN) versus open PN and laparoscopic PN have been reported by large-scale comparative studies and meta-analyses [3,4,5]. Moreover, for highly complex tumors with a Preoperative Aspects and Dimensions Used for an Anatomical score ≥10, Raheem et al. recently reported successful long-term oncological and functional outcomes of RAPN [6].

Successful surgery in RAPN, especially for highly complex tumors, relies on a detailed understanding of the anatomical relations of the tumor absolute and relative to the urinary tract and the vascular structures, including the renal pedicle. Intraoperative navigation with accurate information regarding tumor position relative to the surrounding urinary vascular structures undoubtedly assists the surgeon during RAPN.

Ultra-high-resolution CT (UHR-CT), which constructs 3D images for intraoperative navigation, was recently clinically implemented and investigated. UHR-CT features three different scan modes: normal resolution (0.5 mm × 80 rows/896 channels), high-resolution (HR; 0.5 mm × 80 rows/1792 channels), and super-high-resolution (0.25 mm × 160 rows/1792 channels); the improved spatial resolutions of UHR-CT have been reported by several investigators (Supplementary Figure S1) [7,8,9,10,11,12,13,14,15,16,17,18]. UHR-CT also enables the use of larger matrix sizes (e.g., 1024) in selected CT examinations in routine clinical practice [7,10,13,14,15,17].

**Figure 1:**
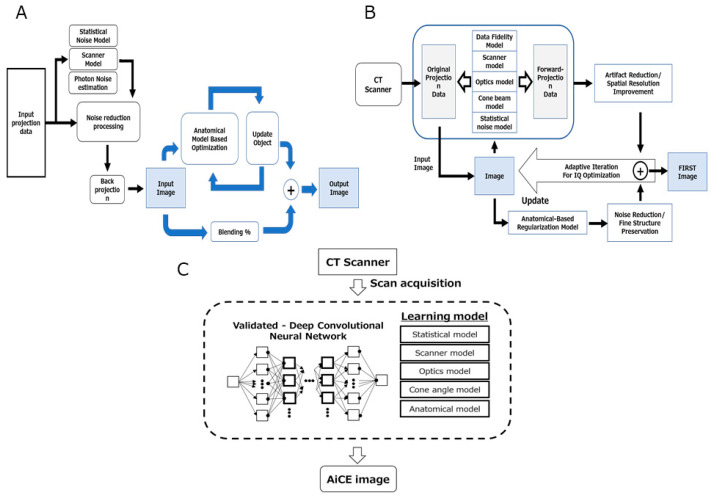
Schematic diagram of the image reconstruction methods. (**A**) Adaptive Iterative Dose Reduction 3D; (**B**) Forward-projected model-based Iterative Reconstruction SoluTion; (**C**) Advanced intelligent Clear-IQ Engine.

With the increasing clinical application of UHR-CT, image noise reduction and signal-to-noise ratio improvements have become important in routine clinical practice. Therefore, Canon Medical Systems has produced hybrid-type iterative reconstruction (Adaptive Iterative Dose Reduction 3D (AIDR 3D)), model-based iterative reconstruction (Forward-projected model-based Iterative Reconstruction SoluTion (FIRST)), and deep learning reconstruction (DLR; Advanced intelligent Clear-IQ Engine (AiCE)) products since 2012 (Figure 1) [10,19,20,21,22]. Furthermore, UHR-CT was suggested to have higher potential to improve image quality and vascular structure evaluations than area-detector CT (ADCT) when DLR is applied rather than hybrid-type iterative reconstruction (IR) in abdominal CT [10].

In this study, we performed RAPN with intraoperative navigation using UHR-CT and compared its perioperative and short-term functional outcomes to those of ADCT. This is the first report to evaluate the feasibility and usefulness of UHR-CT for intraoperative navigation during RAPN.

## 2. Materials and Methods

### 2.1. Patient Population

**Figure 2:**
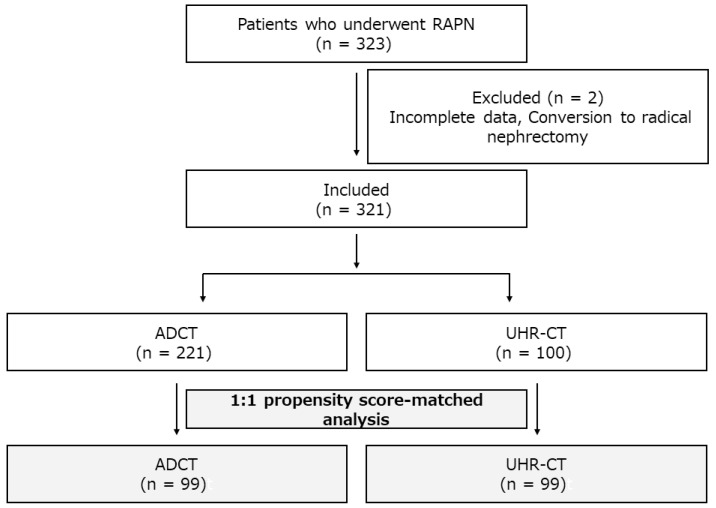
Flowchart of the patients enrolled in the study.

We retrospectively reviewed the clinical data of 323 patients who underwent RAPN at Fujita Health University between July 2010 and July After excluding those for whom incomplete data were available or who required conversion to radical nephrectomy, a total of 321 patients (ADCT: 221; UHR-CT: 100) were enrolled. In the present study, ADCT was used for the initial 221 cases (July 2010~September 2018) and UHR-CT was used for the latter 100 cases (October 2018~July 2020). A 1:1 propensity score–matched analysis was performed, and 99 patients from each group were evaluated (Figure 2).

### 2.2. CT Examination

For the construction of 3D images for intraoperative navigation, all patients underwent unenhanced abdominal CT as well as four-phase dynamic contrast-enhanced (CE) CT examinations using an UHR-CT machine (Aquilion Precision, Canon Medical Systems, Otawara, Japan) or an ADCT (Aquilion ONE, Canon Medical Systems) machine. First, an unenhanced CT image was obtained from the diaphragm to the pelvis during suspended respiration at the end of inhalation. This was followed by a four-phase dynamic CE-CT examination of each CT system.

All patients underwent the placement of an 18–20 G cannula in the antecubital vein before the injection. Preheated iodinated contrast (Iopamiron 370, Bayer Schering Pharma, Osaka, Japan; Iomeprol 350, Eisai Co., Ltd., Tokyo, Japan) was administered by a power injector (Dual Shot GX 7; Nemoto Kyorindo, Tokyo, Japan). The injection dose was 600 mg of iodine per kg of body weight, and the duration was fixed at 18 s, followed by 25 mL of saline solution at the same rate. A bolus tracking program was used to optimize the scanning delay for the renal arterial and venous phases of the dynamic scans. The trigger point was placed at the abdominal aorta at the level of the celiac axis, and the trigger threshold was set at an increase in CT number of more than 100 Hounsfield units over the baseline value. The scan delays were set at 8 s after the trigger, and the renal arterial and venous phases of the dynamic images were obtained serially during a single breath hold. Renal arterial and venous phases of the dynamic scans as the first and second phases were performed from the diaphragm to the lower kidney. The equilibrium phase, as the third phase, was obtained from the diaphragm to the pelvis 90 s after the bolus injection of the contrast media. The excretion phase was scanned as the fourth phase for CT urography from the kidney to the pelvis using CT urography at 300 s after the bolus injection of contrast media.

All unenhanced UHR-CT scans were performed with the following parameters: HR mode, 80 × 0.5 mm collimation, 120 kVp, auto mA with an image noise level of 4, 0.6 s gantry rotation time, 0.813 beam pitch, 512 × 512 matrix, and 320-mm field of view. On the other hand, all unenhanced ADCT scans were performed with the following parameters: 256–320 × 0.5 mm collimation, 120 kVp, auto mA with an image noise level of 11, 0.6 s gantry rotation time, 0.813 beam pitch, 512 × 512 matrix, and 320-mm field of view.

On dynamic CE-UHR-CT, the renal arterial and venous phase scans were performed in HR mode with the following parameters: 80 × 0.5 mm collimation, 120 kVp, auto mA with image noise level of 4, 0.6 s gantry rotation time, 512 × 512 matrix, and 320-mm field of view. The equilibrium and excretion phase scans were performed in the normal resolution mode with the following parameters: 80 × 0.5 mm collimation, 120 kVp, auto mA with an image noise level of 13, 0.6 s gantry rotation time, 0.813 beam pitch, 512 × 512 matrix, and 320-mm field of view.

On dynamic CE-ADCT, the renal arterial and venous, equilibrium and excretion phase scans were performed using the following parameters: 80 × 0.5 mm collimation, 120 kVp, auto mA with an image noise level of 11, 0.6 s gantry rotation time, 0.813 beam pitch, 512 × 512 matrix, and 320-mm field of view.

In this study, the UHR-CT data were reconstructed using filtered back projection between 2010 and 2012, the hybrid-type IR method (AIDR 3D) from 2012 to 2016, the model-based IR method (FIRST) between 2016 and 2019, and the DLR method (AiCE) from 2019 to In contrast, and the ADCT data were reconstructed using hybrid-type IR (AIDR 3D) in each patient during the entire study period.

All CT data obtained by both CT systems were reconstructed as 0.5-mm-thick sections with a standard soft tissue kernel (FC03; Canon Medical Systems) when AIDR 3D was applied in this study. On the other hand, UHR-CT data were reconstructed as 0.5-mm-thick sections by FIRST using body sharp at the standard level (Canon Medical Systems) and AiCE by AiCE body sharp at the standard level (Canon Medical Systems) from 2018 to 2020.

### 2.3. Data Collection

We recorded the following information preoperatively and at 3 and 6 months of follow-up: patient characteristics including age, sex, and body mass index (BMI); clinical disease characteristics including tumor side, surgical approach, RENAL score [23], and presence of a hilar or cystic tumor; and surgical parameters including surgical time, console time, warm ischemia time (WIT), estimated blood loss (EBL), need for blood transfusion, and the presence of complications (Clavien-Dindo) [24]. Hilar tumor was defined as a tumor located in the renal hilum, abutting the renal vessels, and/or the renal pelvis, as observed on the preoperative CT [25,26]. Trifecta achievement was assessed as a composite outcome measure to evaluate the RAPN surgical quality. The definition of trifecta includes a WIT of ≤25 min, no complications, and negative surgical margins [27]. Complications were defined as those with a Clavien-Dindo classification grade of ≥3. The estimated glomerular filtration rate (eGFR; expressed as mL/min/1.73 m^2^), calculated using the Modification of Diet in Renal Disease equation [28], was used to assess renal function.

The study protocol was approved by the Fujita Health University Ethics Review Committee (HM 20-119) and the study was performed in accordance with the ethical standards laid down in the most recent version of the Declaration of Helsinki.

### 2.4. Surgery

All RAPN procedures were performed using a da Vinci Surgical System (Intuitive Surgical, Sunnyvale, CA, USA), as previously described [29]. Briefly, the renal artery or its branches were clamped with a bulldog clamp. The tumor was resected with 2–5 mm of the parenchymal margin. For the inner layer, the collecting system and large vessels were closed with 3-0 V-Loc sutures, and if needed, parenchymal sutures were made with 2-0 V-Loc. The artificial hemostatic sponge TachoSil (Nycomed Austria GmbH, Linz, Austria) was also introduced to increase the coagulation efficiency in the renal capsule.

**Figure 3:**
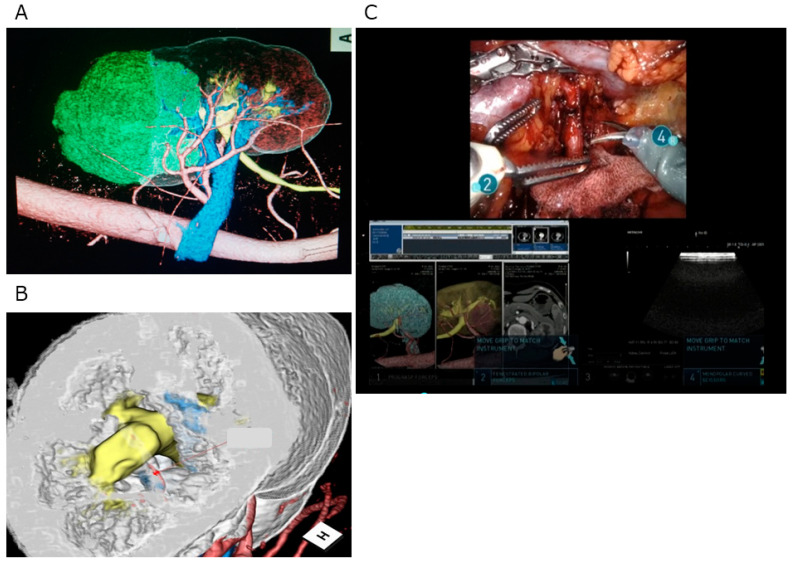
Intraoperative navigation system using ultra-high-resolution computed tomography. (**A**) Image of the tumor, urinary tract, and vascular structures, including the renal pedicle. (**B**) Preoperative image of the cut surface of the tumor. (**C**) Intraoperative navigation system using TilePro software. (**A**) and (**B**) show the same renal tumor; (**C**) shows another renal tumor.

UHR-CT provides clear and accurate images of the anatomical relations of the tumor absolute and relative to the urinary tract and vascular structures, including the renal pedicle. The digital CT data obtained during the early or late artificial and excretory phases were transferred to a workstation (Ziostation 2, version 2.1.x, Qi Imaging, Redwood City, CA, USA) that was used to produce 3D images for intraoperative navigation (Figure 3A). The UHR-CT image data reconstructed on a 3D workstation also provide information regarding the cutting surface, including where vascular stumps are going to be exposed and where the urinary tract is going to open (Figure 3B). All of these data were obtained via an intraoperative navigation system using TilePro software (Figure 3C). All seven surgeons who performed the RAPN had completed the da Vinci certification program approved in Japan.

### 2.5. Statistical Analysis

Owing to inherent differences in baseline patient and disease characteristics between the ADCT and UHR-CT groups, we used 1:1 propensity score–matched analysis to adjust for imbalances in the confounding factors (age, sex, BMI, ASA score, eGFR, tumor side, approach, RENAL score, hilar tumor, and cystic tumor). The propensity scores for each patient were calculated using multivariable logistic regression. A nearest neighbor matching was performed with calipers of width equal to 0.25 of the standard deviation of the logit of the propensity scores. Intergroup comparisons were performed using the Mann–Whitney U-test, chi-square test, or Fisher’s exact test. All data were analyzed using IBM SPSS Statistics version 23 (SPSS Japan Inc., Tokyo, Japan), and *p* values of <0.05 were considered significant in all statistical analyses.

## 3. Results

Among the 323 patients who underwent RAPN at Fujita Health University between July 2010 and July 2020, a total of 321 (ADCT, 221; UHR-CT, 100) were enrolled after exclusions due to incomplete data or conversion to radical nephrectomy. Patient characteristics, including age, sex, BMI, ASA (American Society of Anesthesiology) score, preoperative estimated glomerular filtration rates (eGFR), tumor side, surgical approach, RENAL score, and the presence of hilar or cystic tumors were compared between groups, before and after matching. No significant intergroup differences were observed (Table 1).

Regarding perioperative factors, including surgical time, console time, WIT, EBL, negative surgical margins, pathology, Clavien-Dindo classification ≥3, and trifecta achievement after propensity matching, there were no significant intergroup differences in surgical or console time (ADCT 158 vs. UHR-CT 163, *p* = 0.440; ADCT 110 vs. UHR-CT 112, *p* = 0.483, respectively), whereas the WIT was significantly shorter in the UHR-CT group compared to the ADCT group (15 vs. 17, *p* = 0.032). The EBL was also significantly lower in the UHR-CT group compared to the ADCT group (33 vs. 50, *p* = 0.028). No significant intergroup differences were observed in negative surgical margins (ADCT 100% vs. UHR-CT 99%, *p* = 1.000), clear cell carcinoma in pathology (ADCT 80.8% vs. UHR-CT 72.7%, *p* = 0.246), or complications (ADCT 0% vs. UHR-CT 2.0%, *p* = 0.497). There was also no significant intergroup difference in trifecta achievement (ADCT 80.8% vs. UHR-CT 81.8%, *p* = 1.000) (Table 2).

**Figure 4:**
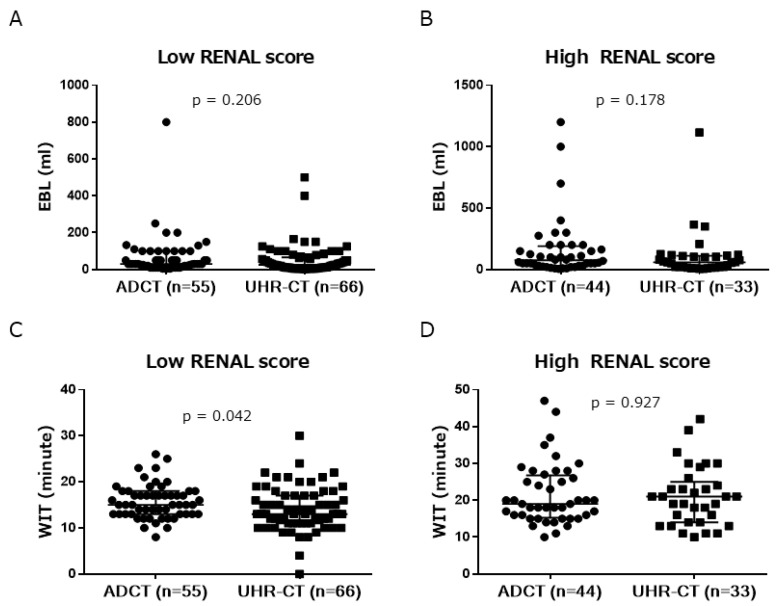
Estimated blood loss (EBL; in mL) of the low (**A**) and high (**B**) RENAL score (ADCT vs. UHR-CT, median with interquartile range) groups. WIT (minutes) of low (**C**) or high (**D**) RENAL score (ADCT vs. UHR-CT, median and interquartile range). ADCT, area-detector computed tomography; UHR-CT, ultra-high-resolution computed tomography.

We next assessed WIT and EBL in the RENAL score category. Each group was divided into the low RENAL score (4–7) or high RENAL score (8–12). The low RENAL score group included 121 patients (ADCT, 55; UHR-CT, 66), while the high RENAL score group included 77 patients (ADCT, 44; UHR-CT, 33). There was no significant difference in EBL in the low or high RENAL score group (ADCT 30 vs. UHR-CT 25, *p* = 0.206; and ADCT 71 vs. UHR-CT 57, *p* = 0.178, respectively) (Figure 4A,B), while the WIT of the UHR-CT group was significantly shorter than that of the ADCT group in the low RENAL score group (ADCT 15 vs. UHR-CT 13, *p* = 0.042) but not in the high RENAL score group (ADCT 19 vs. UHR-CT 21, *p* = 0.927) (Figure 4C,D).

With respect to renal function, there were no significant intergroup differences in the postoperative preservation ratio of eGFR at 3 or 6 months of follow-up (ADCT 91.8% vs. UHR-CT 93.5%, *p* = 0.195; and ADCT 91.7% vs. UHR-CT 94.0%, *p* = 0.160, respectively). (Supplementary Figure S2A,B).

## 4. Discussion

To achieve successful outcomes for RAPN, especially in cases of highly complex tumors, it is necessary to clearly visualize precise information about the key anatomical structures. However, even using recently developed novel augmented-reality imaging techniques [30,31,32], we could not obtain sufficiently precise information for RAPN because ADCT might not provide sufficient image quality. To address these disadvantages, UHR-CT was initiated.

As mentioned above, the reconstructed data in the x-y plane obtained by HR mode with UHR-CT were superior to those obtained by ADCT. In addition, the use of model-based IR and DLR methods to UHR-CT from 2016 to 2019 may have improved its vascular structure visualization over ADCT using the hybrid-type IR method. Therefore, our results are compatible with those of previous studies [10]. Because RAPN success depends on the obtainment of accurate information on the anatomical relationships of the tumor and urinary vascular structures surrounding it, we believe that UHR-CT might be an optimal tool, especially for vascular structure evaluations. UHR-CT was first used to construct 3D images for intraoperative navigation during RAPN.

In this study, perioperative outcomes and renal function at 3 and 6 months after RAPN were compared between the UHR-CT and ADCT groups after propensity score matching. The EBL during RAPN was significantly lower in the UHR-CT group than in the ADCT group. We believe that the EBL was significantly less with intraoperative navigation using UHR-CT because it provided accurate information about the vascular structures, including small vessels such as the segmental renal artery. Furthermore, the median WIT was significantly shorter in the UHR-CT group than in the ADCT group, and both were less than 25 min. Considering our observation that WIT was significantly shorter in the low RENAL score group, RAPN procedures for tumors that were not highly complex were steadily performed with intraoperative navigation using UHR-CT.

Warm ischemic damage is regarded the most important factor affecting postoperative renal function [33]. Therefore, intensive efforts have been made to minimize the damage induced by warm ischemia during PN. UHR-CT image data reconstructed on a 3D workstation indicate, for example, in which direction the tumor should be excised and how deeply it penetrates, allowing for an accurate preoperative simulation of its surface left behind after the excision, including where vascular stumps will be exposed and where the urinary tract will open. We believe that obtaining this accurate information with an intraoperative navigation system using UHR-CT leads to a decreased EBL and WIT versus ADCT. Although there were no significant intergroup differences in the postoperative preservation of eGFR at 3 or 6 months of follow-up, the decrease in EBL and WIT acquired with UHR-CT may benefit future renal function.

This study had several limitations. First, it was a retrospective single-institution study that lacked well-designed analyses. The reconstruction modes used with UHR-CT varied over time, which may have affected our results, and the potential for bias must be considered. Second, to adjust for clinical and demographic heterogeneity, we performed a matched-pair analysis, which resulted in a small sample size. Third, although all our surgeons have sufficient experience in performing RAPN, their technical proficiencies may have varied.

## 5. Conclusions

Here we developed an intraoperative navigation system that produces accurate image data using a 3D workstation. Although no differences in short-term renal function were observed, the UHR-CT resulted in shorter WIT and reduced EBL than ADCT in this propensity score–matched cohort. To the best of our knowledge, this is the first report to demonstrate the feasibility and usefulness of UHR-CT for intraoperative surgical navigation during RAPN.

## Figures and Tables

**Table 1:** Patients’ clinical characteristics.

	Pre-Matching			Post-Matching		
Median (IQR) or n (%)	ADCT (n = 221)	UHR-CT (n = 100)	*p* Value	ADCT (n = 99)	UHR-CT (n = 99)	*p* Value
Age	60 (49–68)	62 (54–70)	0.072	62 (50–70)	62 (54–70)	0.444
Sex (%): Male	166 (75.1)	72 (72.0)	0.583	73 (73.7)	71 (71.7)	0.873
Female	55 (24.9)	28 (28.0)		26 (26.3)	28 (28.3)	
BMI, kg/m^2^	24 (22–26)	24 (22–26)	0.334	24 (21–26)	23 (22–26)	0.175
ASA score	2 (1–2)	2 (1–2)	0.274	2 (1–2)	2 (1–2)	0.365
eGFR, mL/min/1.73 m^2^	70 (59–80)	68 (56–80)	0.333	71 (58–80)	68 (56–80)	0.421
Tumor side: Right	114 (51.6)	53 (53.0)	0.904	54 (54.5)	53 (53.5)	0.887
Left	107 (48.4)	47 (47.0)		45 (45.5)	46 (46.5)	
Approach:Transperitoneal	118 (53.4)	47 (47.0)	0.335	57 (57.6)	47 (47.5)	0.203
Retroperitoneal	103 (46.6)	53 (53.0)		42 (42.4)	52 (52.5)	
RENAL score	7 (5–8)	7 (5–8)	0.375	7 (6–8)	7 (5–8)	0.133
Hilar tumor	48 (21.7)	14 (14.0)	0.127	21 (21.2)	14 (14.1)	0.264
Cystic tumor	36 (16.3)	11 (11.0)	0.237	16 (16.2)	11 (11.1)	0.408

**Table 2:** Patients’ surgical outcomes.

	Post-Matching		
Median (IQR) or n (%)	ADCT (n = 99)	UHR-CT (n = 99)	*p* Value
Surgical time, min	158 (136–190)	163 (148–190)	0.440
Console time, min	110 (95–144)	112 (91–133)	0.483
WIT, min	17 (14–20)	15 (12–21)	0.032
EBL, ml	50 (20–104)	33 (10–85)	0.028
Transfusion	3 (3.0)	1 (1.0)	0.621
Negative surgical margins	99 (100)	98 (99.0)	1.000
Pathology, clear cell carcinoma	80 (80.8)	72 (72.7)	0.246
Clavien-Dindo ≥3	0 (0)	2 (2.0)	0.497
Trifecta	80 (80.8)	81 (81.8)	1.000

## Data Availability

No new data were created or analyzed in this study. Data sharing is not applicable to this article.

## Supplementary Materials

The following supporting information can be downloaded at: https://www.mdpi.com/article/10.3390/cancers14082047/s1, Figure S1: Schematic demonstrating the difference in detector architecture between ultra-high-resolution computed tomography (CT) and area-detector CT, Figure S2: Preservation ratio of postoperative eGFR at 3 or 6 months (ADCT vs. UHR-CT, median with interquartile range).
