# Supplementary material for: Novel Intraoperative Navigation Using Ultra-High-Resolution CT in Robot-Assisted Partial Nephrectomy
Source: Cancers (Basel). 2022 Apr 18;14(8):2047. doi: 10.3390/cancers14082047 (PMC9032210; doi:10.3390/cancers14082047)
Supplement: Supplementary file 1 [file cancers-14-02047-s001.zip › cancers-1666742-supplementary.pdf]

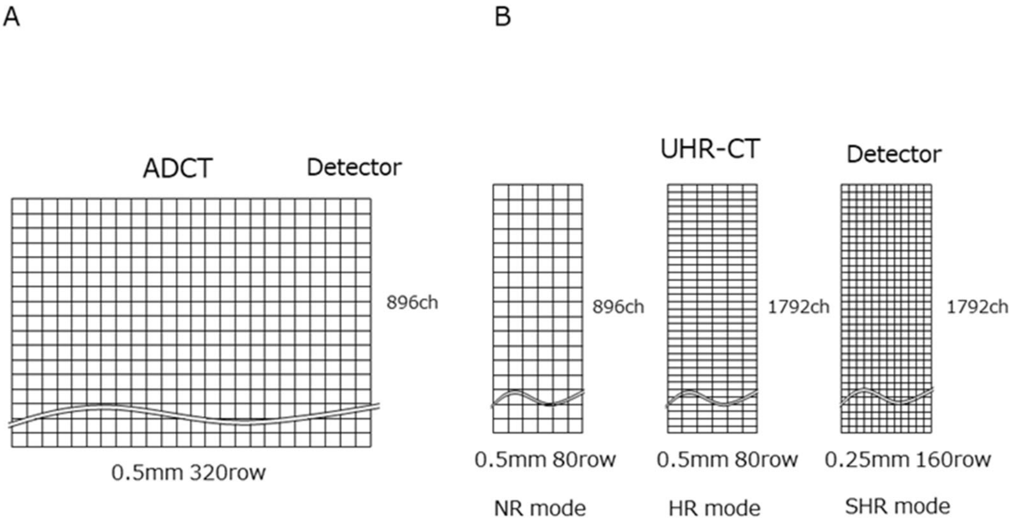

Figure S1. Schematic demonstrating the difference in detector architecture between ultra-high-resolution computed tomography (CT) and area-detector CT. (**A**) Overview of the area-detector CT detector. (**B**) Overview of the ultra-high-resolution CT detector and scan mode.

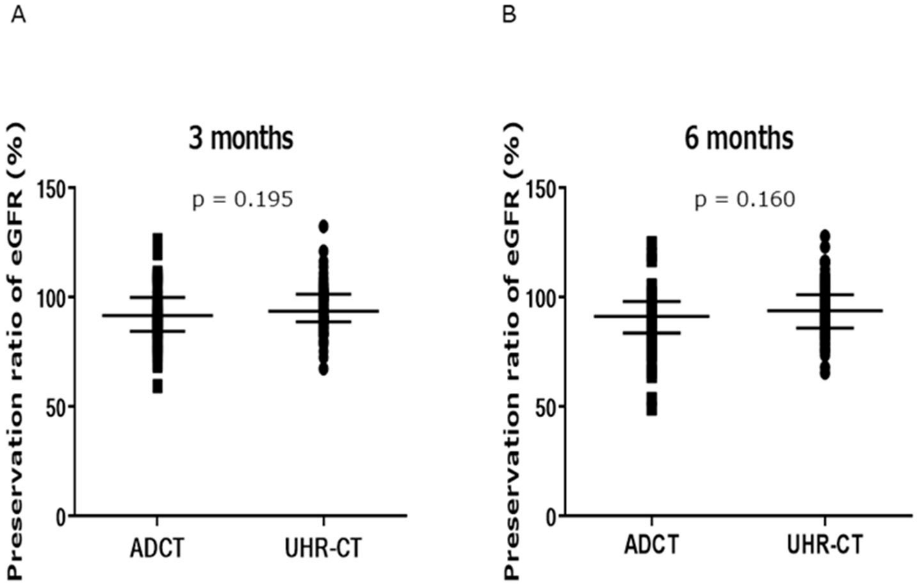

Figure S2. Preservation ratio of postoperative eGFR at 3 or 6 months (ADCT vs. UHR-CT, median with interquartile range). ADCT, area-detector computed tomography; UHR-CT, ultra-high-resolution computed tomography.
